# Fluid responsiveness in septic shock

**DOI:** 10.1186/cc13340

**Published:** 2014-03-17

**Authors:** F Righetti, G Castellano

**Affiliations:** 1 St Boniface Hospital Verona, Italy

## Introduction

The correct assessment of volume status and prediction of response to filling fluid challenge is of crucial importance in the patient with septic shock. A patient is a fluid responder if there is an increase in cardiac index (CI) >15% in response to the fluid challenge. Identifying which patient will be a fluid responder is crucial. The aim of this prospective study is to evaluate which patient is a fluid responder in septic shock using dynamics echocardiography with the distensibility index of the inferior vena cava (DIIVC), which has been shown to be predictive of an increase in CI [1].

## Methods

In a period of 1 year, 42 adult patients were admitted to the ICU in septic shock. All patients were treated according to the guidelines of the Surviving Sepsis Campaign International Guidelines for Management of Severe Sepsis and Septic Shock 2012 [2]. All patients were mechanically ventilated and subjected to transesophageal echocardiography in bicavale projection, calculating the DIIVC through the M-mode by measuring the change in diameter with the acts of positive pressure ventilation and using the formula: (100 × (maximum diameter - minimum diameter)) / minimum diameter. A value >18% was considered a predictor of an increase in CI >15%. The CI was measured continuously through use of the pressure recording analytical method on a Mostcare Vytech monitor. All patients were subjected to a bolus of 500 ml crystalloid through a central venous catheter only after reaching central venous pressure (CVP) ≥10 mmHg during treatment. The results were expressed as the mean with standard deviation and percentage.

## Results

Sixteen patients (38%) had a change in DIIVC <18%, on average 11 ± 5%, and when subjected to fluid challenge did not have an increase in CI >15%, average 8 ± 3%, not proving to be a fluid responder. Twenty- six patients (62%) had a change in DIIVC >18%, on average 45 ± 22%, and when subjected to fluid challenge had an increase of the CI >15%, on average 31 ± 8%, proving to be a fluid responder.

## Conclusion

We have shown that the DIIVC can predict the response of patients to fluid challenge regardless of the values of CVP and then distinguish fluid responders from nonresponder patients. This can help us in the choice of treatment for patients in septic shock.
